# TB far from elimination: an enduring reality in the southeast Asia region

**DOI:** 10.1016/j.lansea.2026.100750

**Published:** 2026-03-13

**Authors:** The Lancet Regional Health – Southeast Asia

Every March, as we mark World TB Day, we confront the enduring reality that tuberculosis (TB)—a disease documented since the dawn of civilisation—still affects a vast population. In our December 2024 Editorial, we highlighted that the world was far from reaching the WHO End TB Strategy milestone of a 50% reduction in TB incidence rates by 2025. Global public health leaders argued that more of the same would not be enough to reach the ambitious target of ending the TB epidemic by 2030, a vision outlined in the Sustainable Development Goals. India, carrying the highest burden of TB in the world, has failed to reach its pledged target of ending TB by 2025, an aspirational goal to be 5 years ahead of the rest of the world in efforts to eliminate TB.

The paradigm shift to active case finding and preventive treatment alongside strengthening active treatment marked a new era in TB elimination efforts. The multipronged strategy of molecular diagnostics, active case finding, contact tracing, and TB infection management, although technically sound, fell short of adequate theoretical grounding or operational clarity. When India renews its efforts to attack TB, it will be prudent to have a population-oriented public health approach—and real-life implementation plan. In this issue of *The Lancet Regional Health – Southeast Asia*, we attempt to bring forth some much-needed evidence to inform policy and implementation perspectives.

Singh and colleagues propose a practical framework for addressing TB in India. The authors argue that elimination of TB built closely on the models of polio and malaria, with strong and intensive efforts in two phases—an intensive reduction in TB cases followed by prevention of disease. The adjustment of interventions by location and intervention package in the framework, which has been extensively applied in malaria control and camp-based service delivery has been widely discussed for TB elimination programmes. Although considered pragmatic by the authors, the framework will still need to be tested and implemented in real-world settings, where there have been historical failures. The implementation will require vast resources and gap analyses to remain sustainable in the long run.

A real-world implementation of a similar approach has been reported by Dorjee and colleagues, among displaced Tibetan communities in refugee settings. The authors report a reduction in TB infection (TBI) incidence in schools where previous screening had taken place. Among people with TBI, the proportion with TB disease was 0.08% (n = 4/4880) in those who received TB preventive treatment (TPT) and 4.4% (n = 95/2144) in those who did not. Followed up over 8 years, children and adolescents who received TPT had a five-fold lower prevalence and 82% lower risk of TB disease (aHR: 0.18; 95% CI: 0.06–0.48) than those who did not receive TPT. Potential of locally driven solutions tailored to communities, using existing tools as highlighted by the authors, is key, as the results of TBI management may also vary between congregate and non-congregate settings. TBI is a dynamic continuum that reversibly progresses between latency and clinical states, which underscores the importance of monitoring TBI and TPT as part of the global strategy to end TB, particularly for children and adolescents.

TPT involves giving anti-TB drugs to individuals with latent infection to eradicate bacilli and prevent progression to active TB. Historically in India, TPT was restricted to two high-risk groups: children under 5 years of age who are household contacts of pulmonary TB patients and people living with HIV. Since 2021, TPT has become a universal preventive strategy under India's National Tuberculosis Elimination Program, with newer short-duration regimens scaled up to include all household contacts of microbiologically confirmed pulmonary TB patients, regardless of age. However, many cases are missed by limited screening and become a silent reservoir capable of sustaining community transmission, a gap widened in resource-constrained settings where non-specialist clinicians may overlook subclinical abnormalities. Timely diagnosis improves cure likelihood and prevents inappropriate TPT, thus narrowing the transmission window. Basu and colleagues argue that this approach optimises resource allocation by avoiding the substantial economic costs of redundant treatment—sequential TPT followed by a full course of anti-tuberculosis treatment after failure—and they advocate adopting a “test-and-treat” model for better returns on investment. However, some of the fundamental questions in screening and management of TBI still remain unanswered and may be better answered through modelling exercises and careful analysis of results of varied implementation protocols in diverse settings.

TB elimination will require a modern, results-driven management mindset. Multisectoral engagement and nutritional policies require local adaptations. Local target setting linked to realistic costing, real-time monitoring of uptake, and structured mid-course correction—standard practices in business and technology—must be integrated into TB control efforts. It is a shame that the world's deadliest infectious disease continues to be managed without such discipline. Until accessibility, accountability, and community ownership are placed at the centre of TB strategy, the promise of “ending TB” will remain largely rhetorical.

